# Effect of Mutation Type on Ectopic Ossification Among Adult Patients With X-Linked Hypophosphatemia

**DOI:** 10.1210/jendso/bvae184

**Published:** 2024-10-22

**Authors:** Hajime Kato, Yasuki Ishihara, Yasuhisa Ohata, Koki Irie, So Watanabe, Soichiro Kimura, Yoshitomo Hoshino, Naoko Hidaka, Yuka Kinoshita, Yuki Taniguchi, Hiroshi Kobayashi, Demetrios T Braddock, Takuo Kubota, Keiichi Ozono, Masaomi Nangaku, Noriko Makita, Nobuaki Ito

**Affiliations:** Division of Nephrology and Endocrinology, The University of Tokyo Hospital, Tokyo 113-8655, Japan; Osteoporosis Center, The University of Tokyo Hospital, Tokyo 113-8655, Japan; Department of Pathology, Yale University, New Haven, CT 06510, USA; Department of Pediatrics, Osaka University Graduate School of Medicine, Suita 565-0871, Japan; The 1st Department of Oral and Maxillofacial Surgery, Osaka University Graduate School of Dentistry, Suita 565-0871, Japan; Department of Cardiovascular Medicine, Osaka University Graduate School of Medicine, Suita 565-0871, Japan; Department of Pediatrics, Osaka University Graduate School of Medicine, Suita 565-0871, Japan; Division of Nephrology and Endocrinology, The University of Tokyo Hospital, Tokyo 113-8655, Japan; Osteoporosis Center, The University of Tokyo Hospital, Tokyo 113-8655, Japan; Osteoporosis Center, The University of Tokyo Hospital, Tokyo 113-8655, Japan; Department of Geriatric Medicine, The University of Tokyo Hospital, Tokyo 113-8655, Japan; Division of Nephrology and Endocrinology, The University of Tokyo Hospital, Tokyo 113-8655, Japan; Osteoporosis Center, The University of Tokyo Hospital, Tokyo 113-8655, Japan; Division of Nephrology and Endocrinology, The University of Tokyo Hospital, Tokyo 113-8655, Japan; Osteoporosis Center, The University of Tokyo Hospital, Tokyo 113-8655, Japan; Division of Nephrology and Endocrinology, The University of Tokyo Hospital, Tokyo 113-8655, Japan; Osteoporosis Center, The University of Tokyo Hospital, Tokyo 113-8655, Japan; Division of Nephrology and Endocrinology, The University of Tokyo Hospital, Tokyo 113-8655, Japan; Osteoporosis Center, The University of Tokyo Hospital, Tokyo 113-8655, Japan; Department of Orthopedic Surgery, The University of Tokyo Hospital, Tokyo 113-8655, Japan; Department of Orthopedic Surgery, The University of Tokyo Hospital, Tokyo 113-8655, Japan; Department of Pathology, Yale University, New Haven, CT 06510, USA; Department of Pediatrics, Osaka University Graduate School of Medicine, Suita 565-0871, Japan; Department of Pediatrics, Osaka University Graduate School of Medicine, Suita 565-0871, Japan; Division of Nephrology and Endocrinology, The University of Tokyo Hospital, Tokyo 113-8655, Japan; Division of Nephrology and Endocrinology, The University of Tokyo Hospital, Tokyo 113-8655, Japan; Osteoporosis Center, The University of Tokyo Hospital, Tokyo 113-8655, Japan; Osteoporosis Center, The University of Tokyo Hospital, Tokyo 113-8655, Japan; Division of Therapeutic Development for Intractable Bone Diseases, Graduate School of Medicine and Faculty of Medicine, The University of Tokyo, Tokyo 113-0033, Japan

**Keywords:** X-linked hypophosphatemia, PHEX, pyrophosphate, ectopic ossification, OPLL

## Abstract

**Context:**

Causative factors for ectopic ossifications in X-linked hypophosphatemia (XLH) remain to be elucidated.

**Objective:**

This work aimed to investigate the genotype-phenotype correlations between the phosphate-regulating endopeptidase homologue, X-linked gene (*PHEX*) and ectopic ossifications in XLH.

**Methods:**

Biochemical data, spinal computed tomography scans, and x-rays of hip/knee joints were retrospectively reviewed. Genetic analysis and the measurement of plasma inorganic pyrophosphate (PP_i_)—a potent inhibitor of tissue calcification—were performed. The effect of *PHEX* mutations on protein function was predicted using nonsense-mediated decay (NMD) and 3-dimensional structure modeling. The index of ossification of the anterior/posterior longitudinal ligament and yellow ligament (OA/OP/OY index) and the sum of the OA/OP/OY index (OS index) were used to quantify the severity of spinal ligament ossification. The severity of the hip/knee osteoarthritis was evaluated by the Kellgren-Lawrence classification.

**Results:**

We examined 24 distinct pathogenic *PHEX* variants in 28 patients from a study population of 33 individuals in 27 unrelated, nonconsanguineous families. Among the 31 patients whose plasma samples were analyzed for PP_i_, 14 patients (45%) showed decreased plasma PP_i_ concentrations; however, PP_i_ concentrations did not correlate with mutation type or ectopic ossification. Fibroblast growth factor 23 levels in women with NMD-insensitive mutations trended lower than in men with NMD-sensitive mutations but failed to reach statistical significance. Both models revealed no correlations between *PHEX* pathogenic variant and ectopic ossification.

**Conclusion:**

Neither modeling found correlates between PHEX pathogenic variants and ectopic ossification. The effects of PP_i_ on ectopic ossifications in adults with XLH revealed trends that should be investigated with a large sample size.

X-linked hypophosphatemic rickets/osteomalacia (XLH, OMIM 307800) is an inherited metabolic disease resulting from inactivating mutations in the phosphate-regulating endopeptidase homologue, X-linked (*PHEX*) gene, with an incidence of approximately 1 in 20 000 births [1, 2]. Although the precise mechanism remains to be determined, loss-of-function *PHEX* mutations result in fibroblast growth factor 23 (FGF23)-induced hypophosphatemia via inappropriately high expression levels of FGF23 in serum. FGF23 regulates serum phosphate through the inactivation of 1,25-dihydroxyvitamin D and the downregulation of sodium/phosphate cotransporters in the renal proximal tubules [3]. Because chronic hypophosphatemia impairs bone mineralization, XLH patients develop rickets and skeletal deformities such as leg bowing and childhood short stature. Adult XLH patients exhibit a high prevalence of ectopic ossifications in the spinal ligaments, enthesopathies in the Achilles tendons, and osteoarthritis [4, 5]. The ectopic ossifications substantially impair the quality of life of XLH patients, but the mechanism by which XLH induces ectopic ossifications is unknown.

Following the identification of *PHEX* pathogenic variants in XLH, genotype-phenotype correlations using height, disease severity, and FGF23 levels have been conducted [6-8], but no study has investigated the relationship between *PHEX* pathogenic variants and ectopic ossifications in XLH. Moreover, a recent murine study of XLH using the Hyp mouse demonstrated that plasma inorganic pyrophosphate (PP_i_)—a potent inhibitor of calcification—was reduced in the Hyp mice compared to wild-type (WT) siblings, suggesting a potential mechanistic role for PP_i_ in the mineralization phenotype of XLH patients [9].

To further investigate the etiology of ectopic ossification in XLH, we used NMD and 3-dimensional (3D) protein structure modeling to predict the effects of *PHEX* pathogenic variants on protein function. We also correlated *PHEX* genotypes and PP_i_ concentrations with the severity and presence of ectopic ossifications in adult XLH patients to investigate a mechanistic role for PP_i_ and *PHEX* genotype on the XLH mineralization phenotype.

## Materials and Methods

### Participants

The genetic and clinical features of 34 adult patients with XLH who had undergone genetic analysis at The University of Tokyo Hospital from 2015 to 2022 were retrospectively reviewed. Plasma samples for PP_i_ concentrations were collected from patients following written consent. The diagnosis of XLH was made based on at least one of the following criteria: 1) the presence of a known *PHEX* pathogenic variant, or 2) chronic hypophosphatemia concomitant with FGF23 levels of 30 pg/mL or higher as measured by a manual enzyme-linked immunosorbent assay (ELISA) kit (KAINOS Laboratories Inc, RRID: AB_2782966) or fully automated chemiluminescent enzyme immunoassay (CLEIA) (Minaris Medical Co Ltd) [10-12]. The clinical data, radiological evaluation of ectopic ossifications, and the severity of dental diseases in 30 adults with XLH had been previously reported (cases 1-30) [4, 13], focusing on the complication incidence and effects of conventional therapy on ectopic ossifications and dental phenotype. In contrast, in the present study we focus on associations between *PHEX* genotype and ectopic ossifications. All procedures were performed following the ethical standards of the Declaration of Helsinki and approved by the institutional ethical board of The University of Tokyo Hospital (Ref. 2879, 11221, and G10115), with the written informed consent of all participants.

### Clinical Analysis

Clinical data, including treatment regimen, age, therapy, and the biochemical data of nonfasting serum phosphate, and albumin-adjusted calcium and FGF23 were collected from electronic health records. The chemistry tests, including serum phosphate, calcium, and albumin, were measured by a LABOSPECT008 (Hitachi) using the manufacturer's standard reagents. FGF23 was assayed by an FGF23ELISA (Kainos, RRID: AB_2782966) or FGF23 CLEIA (Minaris Medical), to detect only full-length FGF23. The criteria for FGF23-related hypophosphatemia in both assays was a measurement greater than 30 pg/mL [10-12].

### Measurement of Plasma Inorganic Pyrophosphate

Plasma was collected from participants and filtered through a 300-kDa membrane (PALL) via centrifugation to remove platelets, and frozen at −80 °C within 1 hour of blood collection. Samples were not thawed until measurement and used only once. Measurement of plasma PP_i_ was performed using adenosine triphosphate sulfurylase as previously described [14, 15]. The luminescence signal was read by EnSpire Multimode Plate Reader (PerkinElmer) at room temperature. Reference ranges of PP_i_ in healthy children and adolescents using the adenosine triphosphate sulfurylase method was recently reported to be 2360 to 4440 nM, which is similar to standard range previously reported in healthy adults (2000-5000 nM) [16].

### Radiological Analysis

The paraspinal ligament ossification was evaluated with plain computed tomography conducted on the entire spine. As in previous studies [4, 13, 17], the ossification indices of the anterior/posterior/yellow ligament (OA/OP/OY indices) were calculated from computed tomography images. The OP index was defined as the number of ossified lesions in the posterior longitudinal ligament (OPLL) as previously reported [18]. This index was calculated by summing the levels of vertebral bodies and intervertebral discs with OPLL. Similarly, the OA index and OF index were adopted to evaluate the severity of the ossification of the anterior longitudinal ligament (OALL) and the yellow ligament (OYL), respectively, as previously reported [4, 13, 17]. The total ossification index (OS index), determined by the sum of the OA, OP, and OY indices, was used to quantify the severity of paraspinal ligament ossification.

For the assessment of osteoarthritis severity, x-ray images of hip joints and knee joints were used. We used the Kellgren-Lawrence (KL) system to evaluate the severity of osteoarthritis with a 5-grade scale (0-4) [19], with KL grade 0 indicating no evidence of osteophytes, KL grade 1 indicating possible osteophyte lipping, KL grade 2 indicating the presence of osteophyte formation, and KL grade 3 indicating moderate joint space narrowing with osteophyte formation. KL grade 4 was assigned to cases with severely reduced joint space and large osteophyte formation. KL grade 2 is generally accepted to equate to the diagnosis of osteoarthritis. The representative grade of hip and knee joint involvement was based on the higher KL grade of either the right or left side. Radiological evaluation was performed by 2 experienced orthopedic surgeons (H.K. and Y.T.). X-ray images of the lower limbs were also categorized into varus or valgus deformity.

### Genetic Analysis

Analyses of *PHEX* mutations were performed with Sanger sequencing or next-generation sequencing with the MiSeq Sequencing System (Illumina) at the Kazusa DNA Research Institute and subsequent multiplex ligation-dependent probe amplification (MRC-Holland) as reported previously [20, 21]. If genetic testing for *PHEX* was negative, next-generation sequencing on genes implicated in hereditary FGF23-related hypophosphatemia was additionally performed at the Kazusa DNA Research Institute. *PHEX* mutations were then categorized into nonsense-mediated messenger RNA decay (NMD)-sensitive mutations and NMD-insensitive mutations according to the 50 to 55 nucleotide rule [22]. Using this rule, we categorized mutations as NMD-sensitive when a terminal codon existed more than 50 to 55 nucleotides upstream from the last exon-exon junction.

### Three-Dimensional Model of *PHEX* Pathogenic Variants

We predicted the 3D structure of *PHEX* pathogenic variants with trRosetta (https://yanglab.qd.sdu.edu.cn/trRosetta/) [23]. The variant PHEX proteins were then evaluated for their binding capacity to bind zinc ions using the Computed Atlas of Surface Topography of proteins (CASTp) (http://sts.bioe.uic.edu/castp/) [24]. Positioning of the zinc-binding site and structural models of *PHEX* pathogenic variants was performed using BioMetAll [25]. The root mean square deviation (RMSD), a quantitative measure for structural difference in WT and *PHEX* pathogenic variants, was calculated using PyMol (https://pymol.org/2/)

### Statistical Analysis

Genotype-phenotype correlations were evaluated as follows: 1) correlation of phenotype with mutation type and location, and 2) correlation of phenotype with the predicted 3D structure of mutated PHEX protein. In our analysis we assumed that female patients with NMD-sensitive mutations resulted in the expression of only a functional *PHEX* allele (group 1), while female patients with NMD-insensitive mutations result in the mixed expression of both normal and dysfunctional *PHEX* alleles (group 2). We additionally assumed that male patients with NMD-insensitive mutations resulted in the expression of only a *PHEX* pathogenic variant (group 3), and that male patients with NMD-sensitive mutations result in null expression of *PHEX* (group 4). Based on these assumptions, we ranked the XLH phenotype in increasing clinical severity as follows: women with NMD-sensitive mutations (group 1), women with NMD-insensitive mutations (group 2), men with NMD-insensitive mutations (group 3), and men with NMD-sensitive mutation (group 4). In analysis 1 we tested our assumptions by comparing the phenotypes of groups with the expected mildest (group 1) and the most severe (group 4) phenotypes. In analysis 2 we tested whether mutant protein structures were associated with clinical phenotype by assuming that the *PHEX* mutations did not follow the NMD-decay 50 to 55 nucleotide rule, as a previous study reported the possibility that *PHEX* mutations did not follow this rule [26]. Statistically significant differences were determined using the Pearson chi-square and the Mann-Whitney *U* test. The Spearman rank correlation was used to evaluate the association between variables. All analyses were performed using the R statistical package version 3.2.4 (R-Core 2016), with *P* less than .05 taken to be statistically significant.

## Results

### Patients

The participants of the study consisted of 33 individuals from 27 unrelated, nonconsanguineous families clinically diagnosed with XLH (Table 1 and Supplementary Table S1) [27]. Each patient presented with inappropriate elevation of FGF23 levels (>30 pg/mL) with concomitant hypophosphatemia. Mutational analysis of *PHEX* was performed in all patients, revealing 24 distinct mutations in 28 patients (Supplementary Table S2) [27]. Although no *PHEX* mutations were detected in 5 patients (cases 25, 29, 31-33), they were ascribed to a clinical diagnosis of XLH due to the presence of mild rickets beginning in childhood, even though subsequent genetic testing for other genes associated with FGF23-related hypophosphatemia was also negative. Most patients presented with ossification of the paraspinal ligament or osteophytes around the knee or hip joints.

**Table 1.** Characteristics of the study population

	RI, adults	All	Male	Female
N		33	16	17
Age, y		39 (18-72)	41 (18-63)	37 (21-72)
Height, SD		−1.8 (−5.1 to 1.8)	−1.5 (−5.1 to 1.0)	−2.1 (−4.7 to 1.8)
Mutation type				
NMD sensitive, n (%)		7 (21)	4 (25)	3 (18)
NMD insensitive, n (%)		17 (52)	6 (38)	11 (64)
Not applicable, n (%)		4 (12)	3 (19)	1 (6)
Mutation not detected, n (%)		5 (15)	3 (19)	2 (12)
Current treatment				
Burosumab, n (%)		4 (12)	2 (13)	2 (12)
Conventional treatment, n (%)		27 (82)	13 (81)	14 (82)
None, n (%)		2 (6)	1 (6)	1 (6)
Biochemical data				
Serum FGF23, pg/mL	10.0-50.0	94.6 (48.9-2890.0)	118.8 (54.0-1574.5)	80.0 (48.9-2890.0)
Serum phosphate, mg/dL	2.7-4.6	2.1 (1.4-3.7)	2.2 (1.4-3.4)	2.1 (1.6-3.7)
Serum calcium, mg/dL	8.8-10.1	9.0 (7.5-10.8)	9.2 (7.5-10.8)	8.9 (8.5-10.0)
Ectopic ossifications				
OALL, n (%)		21 (64)	11 (69)	10 (59)
OPLL, n (%)		11 (33)	4 (25)	7 (41)
OYL, n (%)		26 (79)	11 (69)	15 (88)
Osteophytes around hip joints, n (%)		32 (97)	15 (94)	17 (100)
Osteophytes around knee joints, n (%)		23 (70)	12 (75)	11 (65)
OA index		2 (0-24)	2 (0-24)	1 (0-15)
OP index		0 (0-15)	0 (0-9)	0 (0-15)
OY index		7 (0-16)	6 (0-10)	8 (0-16)
OS index		12 (0-42)	7 (0-42)	12 (0-38)
Hip KL grade		3 (1-4)	3 (1-4)	3 (2-4)
Knee KL grade		2 (0-4)	2 (0-4)	2 (0-4)
Leg deformity				
Valgus deformity, n (%)		6 (18)	3 (19)	3 (18)
Varus deformity, n (%)		14 (42)	8 (50)	6 (35)

Values are reported as median (range).

Abbreviations: FGF, fibroblast growth factor; KL; Kellgren-Lawrence; NMD, nonsense-mediated messenger RNA decay; OA index, ossification of the anterior longitudinal ligament index; OALL, ossification of the anterior longitudinal ligament; OP, ossification of the posterior longitudinal ligament index; OPLL, ossification of the posterior longitudinal ligament; OS index, ossification index; OY index, ossification of the yellow ligament index; OYL, ossification of the yellow ligament; RI, reference interval.

### Plasma Inorganic Pyrophosphate Levels and Correlation With *PHEX* Genotype and Clinical Presentations

**Figure 1.**
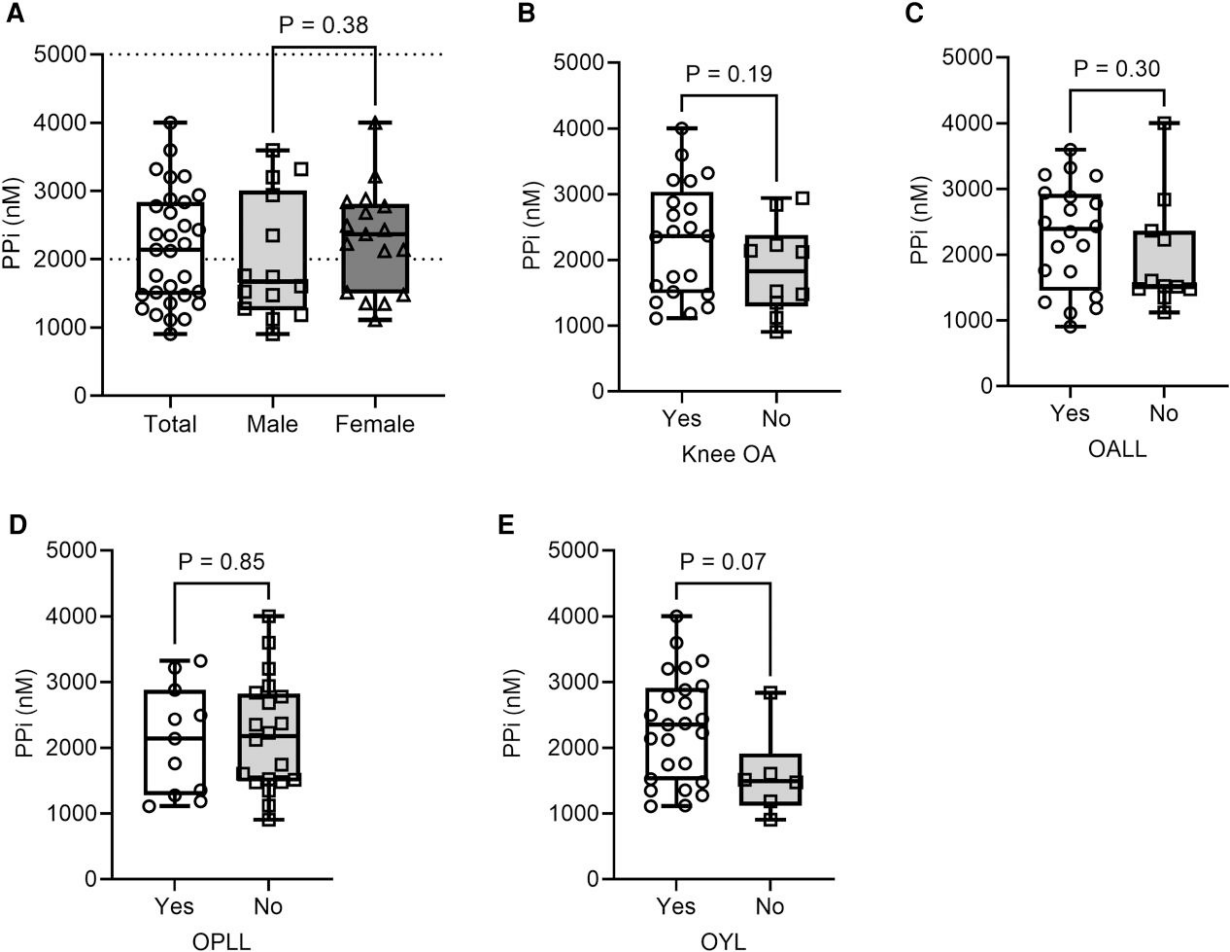
Plasma PP_i_ levels among adult patients with XLH and comparison of plasma PP_i_ levels based on the presence of ectopic ossifications. Plasma PP_i_ levels among all XLH patients and comparison between male and female patients. B to E, Comparison of plasma PP_i_ levels B, based on the presence of knee OA; C, based on the presence of OALL; D, based on the presence of OPLL; and E, based on the presence of OYL. OA, osteoarthritis; OALL, ossification of the anterior longitudinal ligament; OPLL, ossification of the posterior longitudinal ligament; OYL, ossification of the yellow ligament; PP_i_, inorganic pyrophosphate; XLH, X-linked hypophosphatemia. The dot-plot lines show the upper and lower reference range of plasma PP_i_ levels (2000-5000 nM). The median is shown by the line within the box, the 25th and 75th percentiles are box ends, and maximum and minimum values are bar caps. All data are plotted.

Among the 31 patients whose plasma samples were analyzed for PP_i_, 14 (45%) showed decreased plasma PP_i_ (reference range, 2000-5000 nM; Fig. 1; Supplementary Table S1) [16, 27]. There were no statistically significant differences in plasma PP_i_ concentrations in male and female patients (Fig. 1A). No significant correlations were observed between plasma PP_i_ concentrations and the severity and presence of ectopic ossifications (Fig. 1B-1F; Supplementary Table S3A) [27].

### Correlation of Phenotype With Mutation Type and Location

*PHEX* mutations were categorized into NMD-sensitive and -insensitive mutations according to the 50 to 55 nucleotide rule (see Supplementary Table S2) [22, 27]. Seven *PHEX* mutations were categorized as NMD sensitive, and 17 *PHEX* mutations were categorized as insensitive mutations. For 4 mutations (families 2, 6, 7, and 10), including large deletion (family 2), translocation (family 6), and splice site mutations (families 7 and 10), it is difficult to predict the location of the termination codon, which is necessary for evaluation by 50 to 55 nucleotide rules. We, therefore, did not apply 50 to 55 nucleotide rules to these 4 mutations. Comparisons of the clinical data between the 4 groups revealed that FGF23 was highest in male patients with NMD-sensitive mutations (group 4) and lowest in female patients with NMD-sensitive mutations (group 1). Although the observation suggested a correlation between *PHEX* pathogenic variant and serum FGF23, the trend fell short of statistical significance (*P* = .13) (Table 2). There were no differences in height, prevalence of leg deformities, or severity and presence of ectopic ossifications between groups 1 and 4 (see Table 2). As we previously reported that age is the important factor of the ectopic ossifications in patients with XLH [13], subgroup analysis in patients older than 40 years was also performed for the correlation between mutation type and ectopic ossifications, but there was no statistically significant association (data not shown).

**Table 2.** Comparison of biochemical data, ectopic ossifications, and bone deformity between 4 groups

	Female		Male		
	NMD-sensitive (group 1)	NMD-insensitive (group 2)	NMD-insensitive (group 3)	NMD-sensitive (group 4)	*P^a^*
N	3	11	5	5	
Height, SD	−3.1 (−3.2 to −2.6)	−2.0 (−4.7 to 0.7)	−2.5 (−4.2 to −0.3)	−2.5 (−4.2 to −0.3)	.18
Serum FGF23, pg/mL*^b^*	62.4 (48.9-113.8)	87.1 (71.9-1317.8)	100.5 (54.0-158.0)	678.2 (63.5-945.6)	.13
Plasma PP_i_, nM	2433 (2366-2780)	2492 (1476-4000)	1602 (1185-3596)	1757 (1473-2941)	.51
OALL, n (%)	2 (67)	6 (55)	3 (60)	3 (60)	≥.999
OPLL, n (%)	1 (33)	4 (36)	1 (20)	1 (20)	≥.999
OYL, n (%)	3 (100)	9 (82)	3 (60)	3 (60)	.46
Osteophytes around hip joints, n (%)	3 (100)	11 (100)	5 (100)	5 (100)	NA
Osteophytes around knee joints, n (%)	3 (100)	6 (55)	4 (80)	4 (80)	≥.999
OA index	6 (0-15)	1 (0-9)	2 (0-9)	1 (0-22)	.65
OP index	0 (0-14)	0 (0-15)	0 (0-2)	0 (0-9)	.56
OY index	6 (2-9)	8 (0-13)	7 (0-9)	5 (0-10)	.76
OS index	12 (2-38)	14 (0-25)	7 (0-16)	6 (0-41)	.65
Hip KL grade	3 (2-3)	3 (2-4)	3 (2-3)	3 (3-4)	.17
Knee KL grade	3 (2-3)	3 (2-4)	3 (2-3)	3 (3-4)	.64
Varus deformity, n (%)	2 (68)	4 (36)	3 (60)	3 (60)	≥.999
Valgus deformity, n (%)	0 (0)	3 (27)	1 (20)	1 (20)	≥.999

Values are reported as median (range).

Abbreviations: FGF, fibroblast growth factor; KL; Kellgren-Lawrence; NA, not available; NMD, nonsense-mediated messenger RNA decay; OA index, ossification of the anterior longitudinal ligament index; OALL, ossification of the anterior longitudinal ligament; OP, ossification of the posterior longitudinal ligament index; OPLL, ossification of the posterior longitudinal ligament; OS index, ossification index; OY index, ossification of the yellow ligament index; OYL, ossification of the yellow ligament.

^*a*^ *P* value was evaluated between group 1 and group 4.

^*b*^Serum FGF23 levels were compared after excluding patients with estimated glomerular filtration rate lower than 60 mL/min/1.73 m^2^.

### Correlation of Phenotype With Predicted 3-Dimensional Structure of Mutant PHEX Proteins

PHEX is a metalloprotease containing a zinc-binding site [2, 28], and therefore the effect of *PHEX* pathogenic variants on protein function were structurally predicted based on the preservation of the zinc-binding site, as impairment of the metal binding site would impair catalytic function (see Supplementary Table S2) [27]. Additionally, because PHEX has amino acid sequence homology with neprilysin (NEP), a zinc-dependent metalloprotease containing zinc-binding cavity, *PHEX* mutations were additionally categorized based on the presence or absence of the NEP cavity (see Supplementary Table S2) [27]. However, the preservation of both zinc-binding and the NEP cavity failed to correlate with the severity of the XLH clinical phenotype (Tables 3 and 4), and statistically significant differences were not observed by evaluating even potential additive effects of the zinc-binding site and cavity conservation (Table 5). Finally increased RMSD differences between Cα backbone atoms of PHEX pathogenic variants and PHEX WT protein also did not correlate with the severity of ectopic ossifications (Supplementary Table S3B) [27]. Subgroup analysis with patients older than 40 years was also performed for the correlation of the predicted 3D structure and ectopic ossifications, showing no statistical significance (data not shown).

**Table 3.** Comparison of biochemical markers, ectopic ossifications, and leg deformity with and without PHEX zinc binding site

	With zinc binding site (n = 18)	Without zinc binding site (n = 6)	*P*
Height, SD	−2.3 (−4.8 to 0.7)	−2.3 (−3.2 to −0.5)	.79
Serum FGF23, pg/mL*^a^*	94.3 (54.0-1317.8)	113.8 (48.9-945.6)	.74
Plasma PP_i_, nM	2288 (1185-4000)	2399 (1474-2780)	.80
OALL, n (%)	11 (61)	3 (50)	.67
OPLL, n (%)	6 (33)	1 (17)	.63
OYL, n (%)	14 (78)	4 (66.7)	.62
Osteophytes around hip joints, n (%)	19 (100)	6 (100)	≥.999
Osteophytes around knee joints, n (%)	11 (61)	6 (100)	.13
OA index	2 (0-22)	3 (0-15)	.89
OP index	0 (0-15)	0 (0-14)	.56
OY index	8 (0-13)	4 (0-9)	.33
OS index	12 (0-41)	7 (0-38)	.46
Hip KL grade	3 (2-4)	3 (2-4)	.66
Knee KL grade	2 (0-4)	3 (2-3)	.41
Varus deformity, n (%)	8 (44)	4 (67)	.64
Valgus deformity, n (%)	5 (28)	0 (0)	.28

Values are reported as median (range).

Abbreviations: FGF, fibroblast growth factor; KL; Kellgren-Lawrence; OA index, ossification of the anterior longitudinal ligament index; OALL, ossification of the anterior longitudinal ligament; OP, ossification of the posterior longitudinal ligament index; OPLL, ossification of the posterior longitudinal ligament; OS index, ossification index; OY index, ossification of the yellow ligament index; OYL, ossification of the yellow ligament.

^*a*^Serum FGF23 levels were compared after excluding patients with estimated glomerular filtration rate lower than 60 mL/min/1.73 m^2^.

**Table 4.** Comparison of biochemical markers, ectopic ossifications, and leg deformity with and without PHEX cavity

	With cavity (n = 15)	Without cavity (n = 9)	*P*
Height, SD	−1.8 (−4.5 to 0.7)	−2.6 (−4.8 to −1.3)	.18
Serum FGF23, pg/mL*^a^*	97.6 (54.0-1317.8)	83.5 (48.9-1574.5)	.37
Plasma PP_i_, nM	2420 (1185-4000)	2296 (1474-2941)	.54
OALL, n (%)	9 (60)	5 (56)	≥.999
OPLL, n (%)	5 (33)	2 (22)	.67
OYL, n (%)	11 (73)	7 (78)	≥.999
Osteophytes around hip joints, n (%)	14 (100)	9 (100)	≥.999
Osteophytes around knee joints, n (%)	10 (67)	7 (78)	.67
OA index	2 (0-9)	1 (0-22)	.73
OP index	0 (0-15)	0 (0-14)	.74
OY index	7 (0-13)	6 (0-10)	.83
OS index	12 (0-25)	9 (0-41)	.74
Hip KL grade	3 (2-4)	3 (2-4)	.53
Knee KL grade	2 (0-4)	2 (1-4)	.65
Varus deformity, n (%)	6 (40)	6 (67)	.40
Valgus deformity, n (%)	4 (27)	1 (11)	.62

Values are reported as median (range).

Abbreviations: FGF, fibroblast growth factor; KL; Kellgren-Lawrence; OA index, ossification of the anterior longitudinal ligament index; OALL, ossification of the anterior longitudinal ligament; OP, ossification of the posterior longitudinal ligament index; OPLL, ossification of the posterior longitudinal ligament; OS index, ossification index; OY index, ossification of the yellow ligament index; OYL, ossification of the yellow ligament.

^*a*^Serum FGF23 levels were compared after excluding patients with estimated glomerular filtration rate lower than 60 mL/min/1.73 m^2^.

**Table 5.** Evaluation of the additive effect of the zinc-binding site and cavity conservation

	Without zinc-binding site and cavity (n = 5)	With zinc-binding site and cavity (n = 14)	*P*
Height, SD	−2.6 (−3.2 to −1.3)	−2.2 (−4.5 to 0.7)	.64
Serum FGF23, pg/mL*^a^*	88.1 (48.9 −945.6)	94.6 (54.0-1317.8)	.73
Plasma PP_i_, nM	2399 (1474-2780)	2420 (1185-4000)	.67
OALL, n (%)	3 (60)	9 (64)	≥.999
OPLL, n (%)	1 (20)	5 (36)	≥.999
OYL, n (%)	4 (80)	11 (79)	≥.999
Osteophytes around hip joints, n (%)	5 (100)	14 (100)	≥.999
Osteophytes around knee joints, n (%)	5 (100)	9 (64)	.26
Varus deformity, n (%)	4 (80)	6 (43)	.30
Valgus deformity, n (%)	0 (0)	4 (29)	.53
OA index	5 (0-15)	2 (0-9)	.54
OP index	0 (0-14)	0 (0-15)	.65
OY index	6 (0-9)	8 (0-13)	.58
OS index	12 (0-38)	12 (0-25)	.71
Hip KL grade	3 (2-4)	3 (2-4]	.65
Knee KL grade	3 (2-3)	2 (0-4)	.44

Values are reported as median (range).

Abbreviations: FGF, fibroblast growth factor; KL; Kellgren-Lawrence; OA index, ossification of the anterior longitudinal ligament index; OALL, ossification of the anterior longitudinal ligament; OP, ossification of the posterior longitudinal ligament index; OPLL, ossification of the posterior longitudinal ligament; OS index, ossification index; OY index, ossification of the yellow ligament index; OYL, ossification of the yellow ligament.

^*a*^Serum FGF23 levels were compared after excluding patients with estimated glomerular filtration rate lower than 60 mL/min/1.73 m^2^.

### Correlation of Fibroblast Growth Factor 23 Levels With Ectopic Ossifications

Since elevated FGF23 levels are one of the biochemical findings among XLH patients, the correlation between FGF23 level and ectopic ossification was also evaluated. When evaluated among 30 XLH patients after excluding 3 patients with impaired kidney function, lower FGF23 levels were significantly associated with higher OY and OS indexes (Supplementary Table S4A) [27]. However, since age is one of the factors affecting ectopic ossifications and the lower FGF23 group tended to be older than the higher FGF23 group, the subgroup analysis in patients older than 40 was also performed, in which these correlations did not reach statistical significance (Supplementary Table S4B) [27].

## Discussion

Causative factors inducing ectopic ossifications in XLH patients have not been clarified. In the present study we investigated the effect of disrupted PHEX structure and plasma PPi concentrations on the clinical phenotype of XLH patients, specifically focusing on ectopic ossifications. To do so we used 2 independent algorithms to predict the effect of PHEX pathogenic variants on protein function—NMD algorithms to predict allelic expression and 3D structural algorithms to predict the effect of variant sequences on protein function. Both models failed to show statistically significant correlations with adult XLH ossification phenotype. Finally, we measured plasma PP_i_ concentrations, discovering that almost half of the XLH patients exhibited reduced plasma PPi concentrations, but correlations between plasma PP_i_ and ectopic ossifications (entheses in spinal ligaments and osteophytes around hip or knee joint) were not observed.

Previous studies reported *PHEX* to be dose sensitive, but studies analyzing genotype-phenotype correlations in XLH patients have yielded inconsistent results [6-8, 29, 30]. A few studies reported greater disease severity in male patients [29, 30], while others failed to reach sex-specific conclusions [6-8]. Additionally, clinical studies by Holm et al [6] reported that truncation mutations were associated with more severe skeletal phenotypes, and others reported no statistically significant associations of truncating and nontruncating mutations with skeletal phenotype [7, 8, 31]. However, the categories used in previous studies (ie, male and female, truncating and nontruncating) may not have adequately evaluated the effect of PHEX mutations because protein expression may be sex specific as some truncating mutations may not be vulnerable to NMD decay. To address this issue, in our first model we created the 4 groups based on the NMD rule and sex, and compared the clinical data among the expected mildest group 1 (female patients with NMD-insensitive mutations) and group 4, the expected most severely affected group (male patients with NMD-sensitive mutations). Plasma FGF23 levels were noted to be highest in group 4 and lowest in group 1, suggesting a potential effect of *PHEX* genotype on XLH disease severity, but this trend fell just short of statistical significance (*P* = .13). The findings suggests that statistically significant correlations of XLH genotype with disease severity evaluated using NMD algorithms may be established by increasing patient numbers. However, we note that the results are consistent with past studies reporting no association of XLH genotype with clinical phenotype [6-8].

The mechanism by which PHEX regulates FGF23 is undetermined. PHEX is a cell surface endopeptidase, and FGF23 was initially thought to be regulated by PHEX endopeptidase activity [32], but subsequent studies failed to support this hypothesis [33, 34]. Recently, Ishihara et al [8] reported a correlation between the conservation of PHEX zinc-binding and FGF23 levels using 3D structure modeling, but we failed to confirm this observation. The discrepant results may be partly due to the fact that PHEX regulates phosphate homeostasis differently in children and adult XLH patients, as suggested by the murine study of Michigami et al [35]. A further complicating factor may be the observation that FGF23 levels among adult XLH patients are susceptible to variations in renal function [36].

While all previous studies evaluated genotype-phenotype correlations by assessing the severity of bone deformity, height, and disease onset, there has been no evidence of an association between *PHEX* pathogenic variant and adult comorbidities, including paraspinal ligament ossifications and osteoarthritis [6-8], despite the well-known fact that adult XLH patients exhibit a high prevalence of ectopic ossifications although the underlying mechanism is unknown [4, 5, 13, 37]. Thus far, age and severity of dental complications have been significantly associated with the presence and severity of ectopic ossifications [13, 38], and elevated inflammatory markers have been proposed as a potential mechanism for ectopic ossifications [4, 5, 13]. Our study investigated the association of ossification severity and PHEX genotype, failing to find correlations using a structure prediction model predicted on the preservation of the zinc-binding site in the metallo-enzyme. Our findings suggest that the preservation of PHEX enzymatic activity may not be responsible for suppressing ossification in adult XLH patients, and perhaps catalytically independent mechanism are responsible. Increased RMSD between PHEX WT and pathogenic variants also did not correlate with ectopic ossification severity, further supporting the notion that endopeptidase activity may not be driving the mineralization phenotype. In this regard, as the murine homologue of XLH (Hyp mice) has enhanced FGFR signaling in bone [39], dysregulation of FGFR signaling by PHEX impairment may be a potential mechanism for further investigation.

The role of PP_i_ in XLH has never been investigated. A previous study using Hyp mice reported higher PP_i_ concentrations in the bones of Hyp mice [40, 41], suggesting that the mineralization impairment in XLH may result from PP_i_'s role as a potent inhibitor of hydroxyapatite. In contrast, a second study found significantly reduced plasma PP_i_ concentrations in Hyp mouse plasma, suggesting that ectopic ossification in XLH may result from the low plasma PP_i_ levels [9]. Clinical data on the plasma levels of PP_i_ in XLH patients are lacking, and we therefore measured plasma PP_i_ levels of adult XLH patients, finding that almost half the patients exhibited reduced PP_i_ concentrations compared with the normal reference range [16], findings that concur with those of Maulding et al [9]. However, plasma PP_i_ levels in XLH patients did not correlate with paraspinal ligament ossifications and knee and hip osteoarthritis, suggesting that additional factors may be involved.

The limitations of this study include confounding factors such as patient age and treatment history, which significantly affect the timing and severity of XLH enthesopathy [4]. To overcome the possibility that age might affect the actual difference, we tested the subgroup analyses with patients older than 40, but no statistical significance was observed. Also, since treatment histories and regimens are not uniform in this cohort, treatment may have a heterogeneous effect on the clinical presentation. Second, the predictive structural models employed in our structural algorithm may not have accurately captured mutant PHEX structures. Amino acid substitutions in the PHEX active site and the inner surface of the NEP have been reported to dramatically alter the 3D PHEX structure [42, 43], leading us to suspect some pathogenic *PHEX* mutations may have altered protein structures more severely than predicted by our models. Additionally, we did not evaluate the effect of the pathogenic variants on cellular trafficking and endopeptidase activity [44], which may also dramatically affect protein expression. Finally, although we did not observe any statistically significant correlation, that may be due to our small sample size.

In conclusion, we investigated genotype-phenotype correlations between *PHEX* pathogenic variants and XLH disease phenotype, also examining the role of plasma PP_i_ on ossification severity in adult XLH patients. We found that, although PP_i_ concentrations were significantly reduced in XLH patients, they did not correlate with XLH disease severity, and while FGF23 levels were significantly higher in NMD models expected to be more severe, the levels were just shy of statistical significance. Although the trends in plasma PP_i_ and FGF23 levels provide a rationale for understanding the role of *PHEX* variants in XLH severity, establishing clear genotype-phenotype correlations may require increased patient enrollment to enhance the statistical power of the algorithms used in our study.

## Data Availability

Some or all data sets generated during and/or analyzed during this study are not publicly available but are available from the corresponding author on reasonable request.

## Abbreviations

3D: 3-dimensional
FGF: fibroblast growth factor
Hyp: murine homologue of XLH
KL: Kellgren-Lawrence
NEP: neprilysin
NMD: nonsense-mediated decay
OA index: ossification of the anterior longitudinal ligament index
OALL: ossification of the anterior longitudinal ligament
OP index: ossification of the posterior longitudinal ligament index
OPLL: ossification of the posterior longitudinal ligament
OS index: ossification index
OY index: ossification of the yellow ligament index
OYL: ossification of the yellow ligament
*PHEX*: phosphate-regulating endopeptidase homologue, X-linked gene
PP_i_: plasma inorganic pyrophosphate
RMSD: root mean square deviation
WT: wild-type
XLH: X-linked hypophosphatemia
